# Disparities in Obesity Rates among Adults: Analysis of 514 Districts in Indonesia

**DOI:** 10.3390/nu14163332

**Published:** 2022-08-14

**Authors:** Dumilah Ayuningtyas, Dian Kusuma, Vilda Amir, Dwi Hapsari Tjandrarini, Pramita Andarwati

**Affiliations:** 1 Health Policy and Administration Department, Faculty of Public Health, Universitas Indonesia, Depok 16424, Indonesia; 2 Centre for Health Economics & Policy Innovation, Imperial College Business School, London SW7 2AZ, UK; 3 Research Center for Public Health and Nutrition, National Research and Innovation Agency, Bogor 16915, Indonesia; 4 Research Center for Pre-Clinical and Clinical Medicine, National Research and Innovation Agency, Bogor 16915, Indonesia

**Keywords:** obesity, disparity, geographic, socioeconomic, Indonesia

## Abstract

Background: Globally, it was estimated that over 650 million adults 18 years old and older were obese in 2016. It is an increasing global health challenge with a significant health and economic impact. Thus, understanding geographic and socioeconomic disparities in obesity among adults is crucial. Methods: We combined geospatial and quantitative analyses to assess the disparity in obesity across 514 districts in Indonesia. We used the Basic Health Survey (Riskesdas) 2018 for obesity data and the World Bank database for socioeconomic data. Dependent variables included obesity prevalence among all adults (18+ years), males, females, young adults (18–24 years), adults (25–59 years), and older adults (60+ years). Results: We found significant geographic and socioeconomic disparities in adult obesity in Indonesia. In terms of region, districts in Java and Bali had a significantly higher prevalence of obesity than those in Papua, Maluku, and Nusa Tenggara. Districts in Java had 29%, 32%, 60%, and 28% higher prevalence of obesity among all adults, female adults, young adults, and adults. By income, compared to the poorest ones, most affluent districts had a significantly higher prevalence of obesity; they had a 36%, 39%, 34%, 42%, 33%, and 73% higher prevalence of obesity among all adults, males, females, young adults, adults, and older adults. Similarly, by education, compared to the least educated ones, the most educated districts had a significantly higher prevalence of obesity; they had a 34%, 42%, 29%, 36%, and 80% higher prevalence of obesity among all adults, males, females, adults, and older adults. Conclusions: There are significant disparities in adult obesity among 514 districts in Indonesia. Efforts by policymakers and stakeholders are needed to reduce obesity among adults, especially within districts with high prevalence.

## 1. Background

Globally, the World Health Organization (WHO) estimated that over 650 million adults 18 years old and older, or 13% of the world’s adults, were obese in 2016 [1]. It is an increasing global health challenge with significant health and economic impact [2]. Obesity is among the main risk factors for non-communicable diseases, such as cardiovascular diseases and diabetes mellitus, and the leading causes of death and disability in 2019 [1,3]. It is also a major risk factor for musculoskeletal disorders, such as osteoarthritis, and cancers, such as breast, ovarian, and prostate [1]. Economically, recent estimates of eight countries showed the costs of obesity per capita ranged from US$17 in India to US$940 in Australia in 2019—comparable to 1.8% of the gross domestic product (GDP) on average. This economic impacts of obesity are projected to grow to 3.6% of GDP on average by 2060 if there are no significant changes to the status quo [2].

In Indonesia, a lower-middle-income country with a population of over 273 million, the burden of obesity is increasing. The latest Basic Health Survey (Riskesdas) showed the prevalence of obesity (using body mass index of 27 and over) among adults aged 18 years and older more than doubled from 10.5% in 2007 to 21.8% in 2018 [4,5]. Moreover, the latest Global Burden of Study 2019 showed that high body mass index (including obesity) was among the top five risk factors driving the most death and disability in Indonesia, along with other main risk factors for non-communicable diseases such as hypertension, tobacco, dietary risks, and high blood glucose [6].

The linkage between socioeconomic indicators and obesity among adults has been well-studied. For instance, Jaacks et al. [7] proposed the obesity transition framework (using a body mass index of 30 and over) and characterized many countries in Asia, Sub-Saharan Africa, and Latin America in Stages 1 and 2, with a higher prevalence of obesity in women than in men and those with higher socioeconomic status than in those with lower socioeconomic status. On the contrary, most countries in Europe and North America are in Stages 3, with a higher prevalence of obesity among those with lower socioeconomic status [7]. The linkages align with previous studies from low- and middle-income countries (LMICs) [8,9,10,11,12] and high-income countries [13,14,15]. Moreover, previous studies have provided some evidence on geographic disparity in obesity among adults. Slack et al. analyzed data from 3109 counties in the United States and showed that high-obesity regions were concentrated in disadvantaged areas and low-obesity regions were located in more affluent areas [16]. Another study in the United States analyzed data across 74 ZIP code areas within King County (Washington state) and found that obesity rates by ZIP code and median house values were inversely associated [17]. While income and education have been the focus of health disparities research, there is a growing emphasis to better understand geographic disparities to improve health policies that target the population groups that are most vulnerable and with the greatest need [8].

Effective responses to help reduce disparities in obesity are crucial in achieving reduced premature deaths from non-communicable diseases, one of the Sustainable Development Goals [18]. However, current literature on geographic and socioeconomic disparities in obesity among adults have at least two limitations. First, most analyses to examine the socioeconomic disparity used data at the individual level. They include analyses from Nepal and Indonesia in Asia, from Chad and South Africa in Africa, and from Mexico and Colombia in Latin America [8,9,10,11,12]. While such evidence is essential, analyses using locality-level data (such as counties and districts) are also crucial for policies. This is especially relevant in decentralized settings where many health sector policies are transferred to the local level. Second, previous studies on geographic disparity are mainly from high-income countries, especially the United States [16,17]. Such studies in LMICs are limited to analysis using urban/rural instead of other indicators, such as area-level income and education [8,10]. Thus, our analysis aims to assess geographic and socioeconomic disparities in obesity among adults across 514 Indonesian districts.

## 2. Methods

### 2.1. Study Design

We conducted a cross-sectional study to assess geographic and socioeconomic disparities in obesity among adults (18 years and over) across 514 Indonesian districts. For obesity data, we analyzed the latest national health survey RISKESDAS 2018 that was representative at the district level. In terms of sampling, RISKESDAS used two-stage sampling and included 30,000 census blocks and 300,000 households. The first stage selected 30,000 census blocks out of a total of 720,000 census blocks in the country, proportional by urban and rural. The second stage included selecting ten families by employing household head education implicit stratification. For adults, RISKESDAS included 624,563 individuals 18+ years old. Further details on RISKESDAS are provided elsewhere [19,20]. 

### 2.2. Dependent Variables

We used six indicators of obesity as dependent variables: obesity among all adults (18 years and over), male adults, female adults, young adults (18–24 years), adults (25–59 years), and elderly or older adults (60 years and over). Obesity was defined as a body mass index of 27.0 and above, per the Ministry of Health Regulation 41/2014. We assessed the prevalence by sex to see different patterning for males and females, as characterized in the obesity transition framework [7]. We evaluated the prevalence by age category to see the patterning among young adults, adults, and older adults, which is essential for NCD control and prevention and for designing effective health system responses [18]. 

### 2.3. Independent Variables

**Figure 1:**
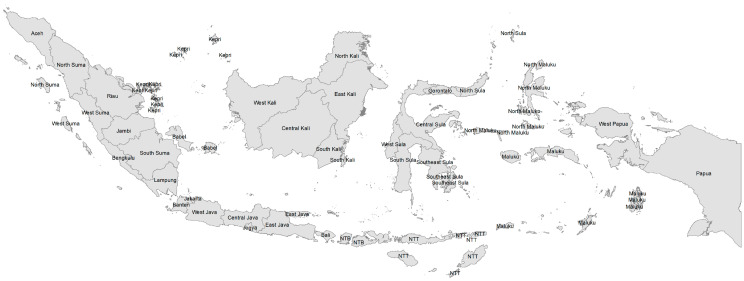
Map of Indonesia by province. Note: Suma = Sumatera, Kepri = Riau Islands, Sula = Sulawesi, Kali = Kalimantan, NTB = West Nusa Tenggara, NTT = East Nusa Tenggara. We divided the provinces into five regions, including Sumatera, Java/Bali, Kalimantan, Sulawesi, and Papua/Maluku/Nusa Tenggara. Java/Bali is the most developed and Papua/Maluku/Nusa Tenggara is the least developed. We obtained the shapefile from the Indonesian Information and Geospatial Agency and created the map in ArcMap 10.

For geographic and socioeconomic data, we analyzed data on urban/rural, region, income, and education at the district level available from the World Bank [21]. We defined cities as urban and regencies as rural. We divided the country into five regions, including Sumatera, Java (and Bali), Kalimantan, Sulawesi, and Papua (and Nusa Tenggara and Maluku)—see Figure 1. In terms of development, the western part of the country, especially Java and Bali, is generally the most developed, while the eastern part, especially Papua, Nusa Tenggara, and Maluku, is the least developed [20,22,23]. Using poverty rates at the district level, we divided the districts into five quintiles, with the poorest districts having the highest poverty rates in the first quintile. Using the senior secondary net enrollment ratio, we divided the districts into five quintiles, with the least educated districts having the lowest net enrollment ratio in the first quintile [20,22,23]. 

### 2.4. Data Analysis

We performed geospatial analysis by analyzing the obesity prevalence by provinces and districts. We employed multivariate analysis using Ordinary Least Square to assess the associations between the independent variables (including urban/rural, region, income, and education) and dependent variables (including obesity among all adults, male adults, female adults, young adults, adults, and older adults). We compared the absolute and relative differences between the most developed and least developed regions, poorest and wealthiest districts, and least and most educated districts. We considered the 5% level or lower as statistically significant. Geospatial analysis and multivariate analysis were performed in ArcMap 10 and STATA 15, respectively. 

## 3. Results

### 3.1. Provincial Level Analysis

**Figure 2:**
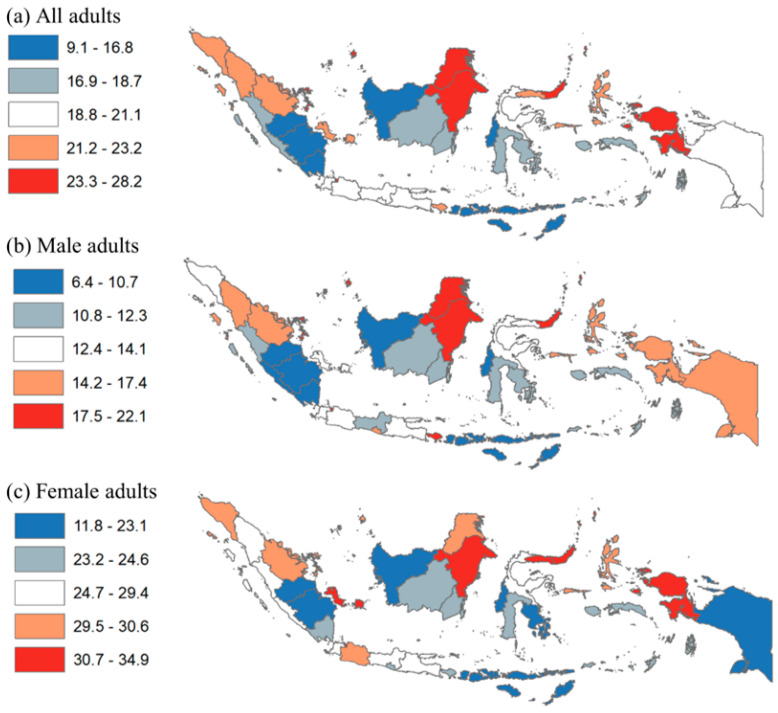

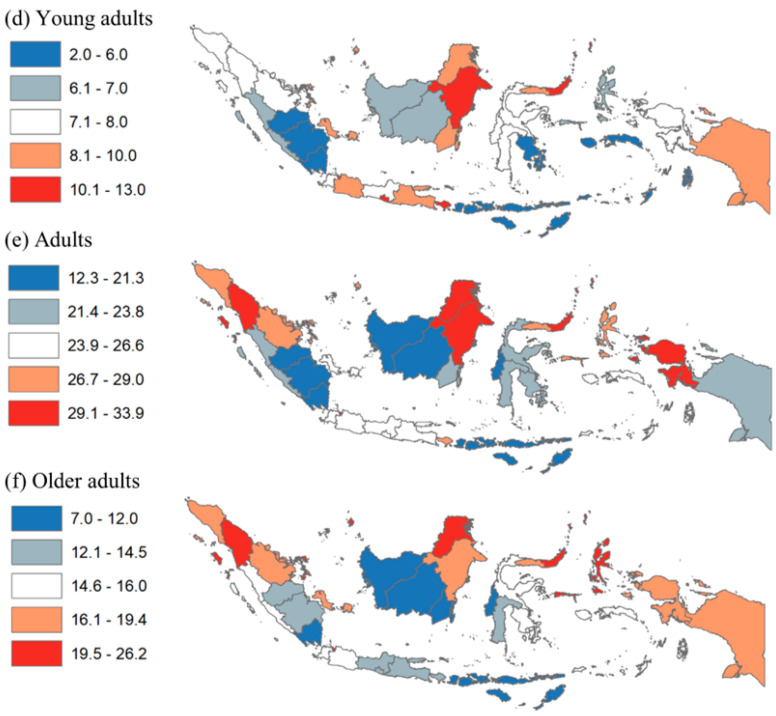
Disparity of obesity among adults by province in Indonesia, 2018. Note: Numbers show prevalence of obesity among all adults, males, females, young adults, adults, and older adults.

For geographic disparities, Figure 2 provides the prevalence of obesity by quintile at the province level. In panel (a), the prevalence of obesity among all adults ranged from 9.1% to 28.2%; that among male adults ranged from 6.4% to 22.1%; that among female adults ranged from 11.8% to 34.9%; that among young adults ranged from 2.0% to 13.0%; that among adults ranged from 12.3% to 33.9%; that among older adults ranged from 7.0% to 26.2%. For all adults, the prevalence of obesity was highest (quintile 5) in Jakarta, Riau Islands, North Kalimantan, East Kalimantan, North Sulawesi, and West Papua. For males, obesity prevalence was highest in Jakarta, Riau Islands, North Kalimantan, East Kalimantan, North Sulawesi, and Bali. For females, the prevalence was highest in Jakarta, Bangka Belitung, East Kalimantan, North Sulawesi, Gorontalo, and West Papua. For young adults, the prevalence was highest in Jakarta, East Kalimantan, North Sulawesi, Yogyakarta, and Bali. For adults, the prevalence was highest in Jakarta, North Sumatera, North Kalimantan, East Kalimantan, North Sulawesi, and West Papua. For older adults, the prevalence was highest in Jakarta, Riau Islands, North Sumatera, North Kalimantan, North Sulawesi, and North Maluku. Note that obesity prevalence in Jakarta and North Sulawesi was highest for all adults, by sex, and by age groups.

For socioeconomic disparities, the prevalence of obesity by income at the province level is shown in Table 1. The top box shows the wealthiest provinces (including Bali, Jakarta, North Kalimantan, and Riau Islands) and the bottom box shows the poorest provinces (including Maluku, East Nusa Tenggara, and West Papua). The grey-shaded prevalence shows higher than the national average in each column. Among the ten wealthiest provinces, five had consistently higher than the national average for every obesity indicator (i.e., Jakarta, North Kalimantan, East Kalimantan, and Riau Islands). In contrast, among the ten poorest provinces, only two did (i.e., Gorontalo and West Papua).

### 3.2. District Level Analysis

The characteristics of districts and obesity among adults are shown in Table 2. Among 514 districts, 97 were cities and 417 were regencies. Out of 514 districts, 30.0% were in Sumatera, while 24.9% were in Java and Bali. Moreover, 79% of urban districts (cities) were wealthier districts in quintiles 4 and 5, and 47.2% of rural districts (regencies) were poorer districts in quintiles 1 and 2. In terms of education, 71.1% of urban districts were the most educated districts in quintiles 4 and 5, and 46.8% of rural districts were the least educated districts in quintiles 1 and 2. Regarding dependent variables (panel b), the prevalence of obesity among all adults was 19.0%; that among males and females was 12.8% and 25.6%; and that among young adults, adults, and older adults was 7.6%, 23.8%, and 14.8%, respectively. The prevalence of obesity was significantly higher in urban areas than in rural areas. Obesity prevalence among all adults in urban areas was higher by 1.35 (i.e., 24.2% divided by 17.9%) compared to that in rural areas. By sex, obesity prevalence among male and female adults in urban areas was higher by 1.54 and 1.25 times, respectively. By age group, obesity prevalence among young adults, adults, and older adults in urban areas was higher by 1.32, 1.33, and 1.82 times, respectively.

**Figure 3:**
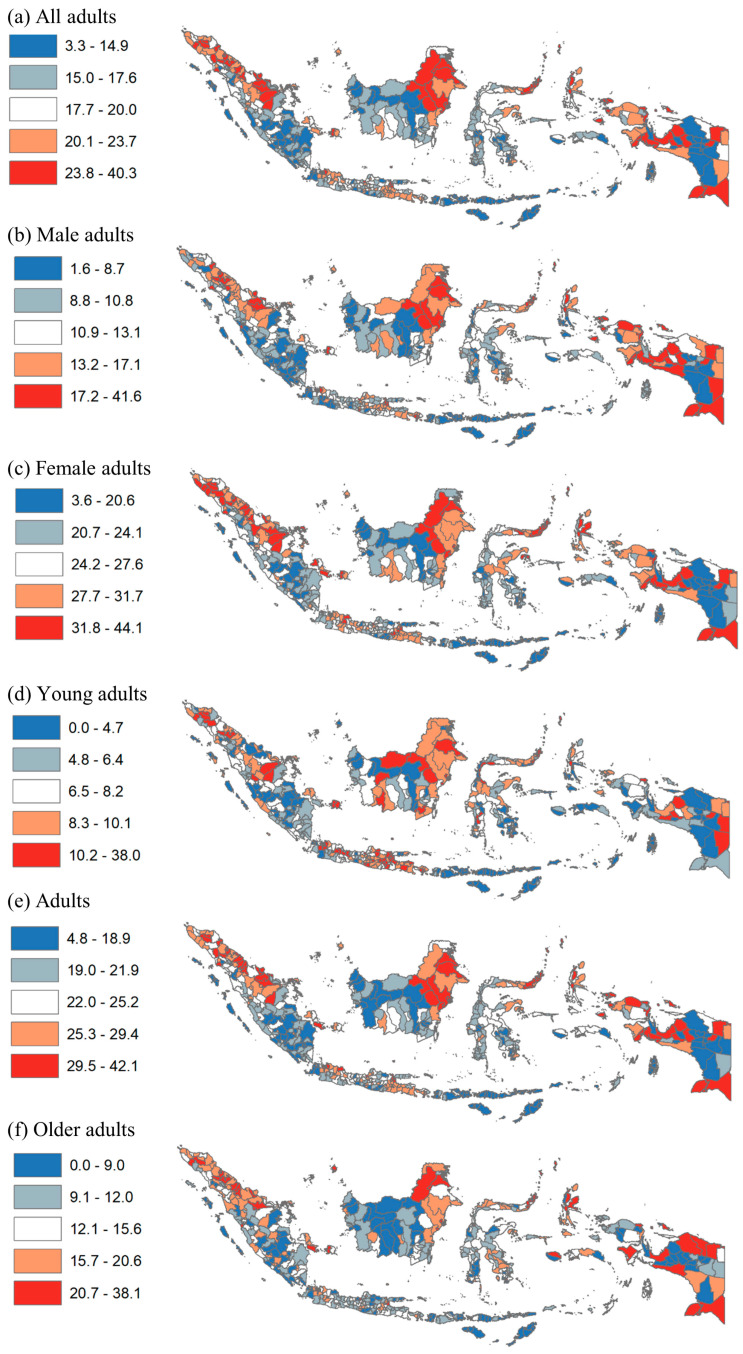
Disparity of obesity among adults by district in Indonesia, 2018. Note: Numbers show prevalence of obesity among all adults, males, females, young adults, adults, and older adults.

For geographic disparities, Figure 3 provides the district-level disparity in obesity by prevalence quintile. Many districts in Aceh, North Sumatera, Riau, East Java, and Papua provinces had the highest prevalence of obesity among all adults. Additionally, many districts in the provinces of Bangka Belitung, West Java, East Java, and Papua had the highest obesity among female adults. Many districts in the provinces of West Papua, Papua, Aceh, and Riau had the highest obesity among young adults. 

For socioeconomic disparities, ten districts with the lowest and highest burden of obesity among adults are shown in Table 3 and Table 4, respectively. For all adults, obesity prevalence ranged from 3.3% in the Sumba Tengah regency (East Nusa Tenggara) to 40.3% in Yalimo (Papua). By sex, obesity among males ranged from 2.0% in Sumba Tengah and Sabu Raijua (East Nusa Tenggara) to 41.6% in Yalimo (Papua); obesity among females ranged from 4.0% in Sumba Barat Daya (East Nusa Tenggara) to 44.1% in Kep. Seribu (Jakarta). By age group, obesity among young adults ranged from 0% in Manggarai Timur and Belu (East Nusa Tenggara) to 38.0% in Yalimo (Papua); that among adults ranged from 4.8% in Sumba Tengah (East Nusa Tenggara) to 42.1% in Padang Sidempuan (North Sumatera); that among older adults ranged from 0% in nine regencies in Papua province to 38.1% in Kota Banda Aceh (Aceh). By urbanicity, almost all districts with the lowest obesity for all adults, by sex, and by age groups are rural, but about half of those with the highest obesity were urban. By income level, poverty rates among the ten districts with the highest obesity averaged up to 18%, while that among the ten districts with the lowest obesity averaged up to 37%. 

We provide the associations between geographic and socioeconomic measures and obesity in Table 5. In terms of region, districts in Java and Bali had a significantly higher obesity prevalence among all adults, female adults, young adults, and adults than those in Papua, Nusa Tenggara, and Maluku. Additionally, districts in Java and Bali had 29%, 32%, 60%, and 28% higher obesity prevalence among all adults, female adults, young adults, and adults, respectively. By income, the wealthiest districts had a significantly higher prevalence of obesity among adults, males, females, young adults, adults, and older adults than the wealthiest districts. Wealthiest districts had 36%, 39%, 34%, 42%, 33%, and 73% higher prevalence of obesity among adults, males, females, young adults, adults, and older adults, respectively. By education, the most educated districts had a significantly higher prevalence of obesity among adults, males, females, adults, and older adults than the least educated districts. Most educated districts had 34%, 42%, 29%, 36%, and 80% higher prevalence of obesity among adults, males, females, adults, and older adults, respectively. These results were similar among rural districts. 

## 4. Discussion

We found a high prevalence of obesity among adults 18 years and older in Indonesia. Using the national obesity cut-off of body mass index 27 and over, the prevalence among all adults, males, and females was 19.0%, 12.8%, and 25.6%, respectively. These findings characterize the country being in Stage 1 of the obesity transition, although further along within the stage, together with other countries with a higher per capita GNI in Southeast Asia, such as the Philippines and Thailand [7]. Moreover, by age, the prevalence among young adults (18–24 years), adults (25–59 years), and older adults (60 years and over) was 7.6%, 23.8%, and 14.8%, respectively. 

We also found significant and large geographic and socioeconomic disparities in obesity among adults across 514 Indonesian districts. By urban/rural, obesity prevalence among all adults, males, females, young adults, adults, and older adults was significantly higher among urban districts (i.e., cities) compared to rural ones (i.e., regencies). These findings align with previous studies in other LMICs, including Nepal, Iran, Chad, and South Africa [8,10,11]. A review by Ford et al. [24] asserted that diet and physical activity are the two main drivers of obesity in LMICs. Urban areas tend to have higher availability of calorie-dense and cheap foods. Additionally, people in urban areas tend to have reduced physical activity through changes in infrastructure, transportation, and occupational activities [24]. However, our analysis also found that while all districts in the bottom ten districts with the lowest prevalence of all obesity indicators were rural, nearly half of the districts in the top ten districts with the highest prevalence were also rural. This may be because some rural districts (e.g., Kab. Karo in North Sumatera or Kab. Minahasa in North Sulawesi) have already had a similar economic development (e.g., income and education level) to nearby urban districts (e.g., Kota Tomohon and Kota Padang Sidempuan). All this indicates that, given limited resources, effective responses to reduce disparity in obesity may prioritize urban districts and rural districts with high obesity prevalence [25,26,27]. 

By region, the prevalence of all obesity indicators was higher in the most developed region (i.e., the Java region including Bali) than in the least developed region (e.g., the Papua region including Maluku and Nusa Tenggara). This finding aligns with previous studies. A study across 3109 counties in the United States found that high-obesity regions were concentrated in disadvantaged areas and low-obesity regions were located in more affluent areas [16]. Globally, obesity prevalence among adults was higher among LMICs with lower national income in Stage 1 of the obesity transition compared to LMICs with higher income in Stage 2 and high-income countries in Stage 3 [7]. 

By income, the wealthiest districts had a significantly higher prevalence of obesity by up to 73% (among older adults) than the poorest districts. By education, the most educated districts had a significantly higher prevalence of obesity by up to 80% (among older adults) than the least educated districts. This finding aligns with previous studies in Nepal, Iran, Chad, South Africa, and other LMICs [8,10,11]. Additionally, it aligns with Stages 1 and 2 of the obesity transition with a higher prevalence of obesity in those with higher socioeconomic status than in those with lower socioeconomic status [7]. 

For policy, obesity is already very high among young adults and adults as the primary working population, which may have an economic impact from lower productivity and increased cost of illness due to obesity-related health issues [2]. Moreover, the high burden of obesity among older adults may indicate the need to reorient the health system to better prevent and control obesity and other risk factors throughout the care continuum, from the community to primary care to secondary/tertiary care, potentially through integration with infectious disease platforms [28,29,30]. By sex, a much higher prevalence among women in Indonesia may be related to maternal obesity, indicating that effective intervention at the population and health systems levels are needed at each stage (e.g., pregnancy and post-partum) [31]. By region and socioeconomic status, given limited resources, effective responses to reduce disparity in obesity may prioritize more affluent urban districts and rural districts with higher burden of obesity and other non-communicable disease risk factors [32,33,34,35]. 

To our knowledge, our analysis is the first on the geographic and socioeconomic disparity in obesity among all adults, males, females, young adults, adults, and older adults using a relatively large number of subnational units (e.g., 514 districts) in LMICs. However, our study has at least two limitations. First, our dataset did not have information on ethnicity and migration, so we were not able to explore sub-group analysis by those variables [36]. Second, using cross-sectional data, our study was limited to assess the trends the obesity prevalence over time. Nonetheless, our findings have important policy implications, especially on the prevention and control of non-communicable diseases in Indonesia and other LMICs.

## 5. Conclusions

In Indonesia, the prevalence of obesity was highest among females (26.4%) and adults aged 25–59 years (24.8%). There were significant geographic and socioeconomic disparities in adult obesity. Compared to districts in the least developed region, those in the most developed region had 29%, 32%, 60%, and 28% higher prevalence of obesity among all adults, females, young adults aged 18–24 years, and adults. Compared to the poorest districts, the most affluent ones had a 36%, 39%, 34%, 42%, 33%, and 73% higher prevalence of obesity among all adults, males, females, young adults, adults, and older adults aged 60+ years. Compared to the least educated districts, the most educated ones had a 34%, 42%, 29%, 36%, and 80% higher prevalence of obesity among all adults, males, females, adults, and older adults. Efforts are needed to reduce obesity among adults, especially within districts with high prevalence.

## Figures and Tables

**Table 1:** Prevalence of obesity among adults by province in Indonesia, 2018.

		Obesity Prevalence					
	Poverty				Young		
	Rates	All	Males	Females	Adults	Adults	Older Adults
	(1)	(2)	(3)	(4)	(5)	(6)	(7)
Bali	4.5%	21.9%	19.2%	24.6%	11.4%	26.7%	14.2%
South Kalimantan	4.8%	17.9%	11.8%	24.3%	8.6%	21.9%	12.0%
Central Kalimantan	5.0%	17.2%	11.2%	24.0%	7.3%	21.3%	10.3%
Jakarta	5.0%	28.2%	22.1%	34.4%	12.3%	32.7%	26.2%
Banten	5.3%	20.3%	13.4%	27.7%	7.6%	25.2%	15.4%
Bangka Belitung	5.4%	22.0%	14.0%	31.1%	9.9%	26.6%	18.5%
West Sumatera	6.6%	18.7%	11.6%	25.8%	6.9%	23.8%	15.3%
North Kalimantan	7.0%	23.8%	17.9%	30.6%	9.2%	29.5%	20.5%
East Kalimantan	7.1%	26.6%	20.3%	33.8%	13.1%	31.8%	19.4%
Riau Islands	7.6%	24.2%	19.0%	29.9%	8.6%	29.0%	20.9%
Jambi	7.8%	16.1%	10.7%	21.9%	5.7%	19.8%	13.8%
North Maluku	7.9%	22.1%	14.3%	30.3%	6.6%	28.2%	21.1%
West Java	7.9%	21.1%	13.1%	29.5%	9.0%	26.4%	15.7%
West Kalimantan	8.1%	15.6%	10.2%	21.3%	6.6%	19.6%	10.7%
North Sulawesi	8.5%	27.9%	21.3%	34.9%	11.4%	33.9%	25.9%
Riau	8.8%	22.1%	15.0%	29.8%	8.0%	27.7%	17.0%
South Sulawesi	9.8%	17.4%	11.2%	23.4%	7.8%	22.1%	12.8%
West Sulawesi	10.3%	16.8%	10.7%	23.1%	7.6%	21.3%	11.6%
East Java	10.9%	20.9%	13.9%	27.9%	10.0%	26.1%	13.6%
Central Java	10.9%	18.9%	12.2%	25.4%	8.5%	23.9%	12.5%
North Sumatera	11.3%	23.2%	17.1%	29.4%	8.4%	29.3%	23.3%
Lampung	12.6%	15.9%	8.7%	23.6%	6.1%	19.8%	11.5%
Yogyakarta	12.7%	20.3%	16.5%	24.0%	10.8%	24.8%	14.5%
Southeast Sulawesi	13.0%	17.3%	11.8%	22.8%	5.2%	22.4%	15.3%
South Sumatera	13.1%	15.9%	9.9%	22.3%	5.5%	20.0%	12.6%
Central Sulawesi	14.6%	19.0%	12.5%	25.9%	7.5%	23.4%	15.4%
West Nusa Tenggara	14.8%	13.5%	7.0%	19.5%	4.4%	17.9%	8.3%
Bengkulu	15.0%	18.3%	10.3%	26.8%	6.8%	22.3%	15.4%
Aceh	16.4%	22.2%	13.9%	30.6%	8.0%	28.3%	17.3%
Gorontalo	16.8%	22.3%	14.1%	30.7%	9.6%	28.0%	17.4%
Maluku	21.8%	17.8%	12.3%	23.4%	4.1%	23.9%	16.0%
East Nusa Tenggara	22.0%	9.1%	6.4%	11.8%	2.5%	12.3%	7.0%
West Papua	26.5%	24.0%	17.4%	31.6%	8.4%	30.1%	18.8%
Papua	29.4%	18.9%	15.1%	23.1%	8.8%	21.7%	17.8%
AVERAGE		19.9%	13.7%	26.4%	8.0%	24.8%	15.8%

Note: Ordered by the average poverty rates (column 1), the provinces in the top box are richest and those in the bottom box are poorest. Shaded values are higher than the national average for each group.

**Table 2:** Characteristics of districts and obesity among adults.

		All		Urban		Rural		Difference	
		n	%	n	%	n	%	%	
		(1)	(2)	(3)	(4)	(5)	(6)	(7) = (4–6)	
(a) Characteristics									
	Sample size district	514	100%	97	100%	417	100%	0%	
	Region								
	Papua	95	18.5%	9	9.3%	86	20.6%	11.3%	
	Java	128	24.9%	35	36.1%	93	22.3%	−13.8%	
	Sumatera	154	30.0%	33	34.0%	121	29.0%	−5.0%	
	Kalimantan	56	10.9%	9	9.3%	47	11.3%	2.0%	
	Sulawesi	81	15.8%	11	11.3%	70	16.8%	5.4%	
		514		97		417			
	Income/poverty								
	Q1 poor	102	19.8%	3	3.1%	99	23.7%	20.6%	
	Q2	103	20.0%	5	5.2%	98	23.5%	18.3%	
	Q3	103	20.0%	13	13.4%	90	21.6%	8.2%	
	Q4	103	20.0%	22	22.7%	81	19.4%	−3.3%	
	Q5 rich	103	20.0%	54	55.7%	49	11.8%	−43.9%	
		514		97		417			
	Education								
	Q1 least	103	20.0%	0	0.0%	103	24.7%	24.7%	
	Q2	103	20.0%	11	11.3%	92	22.1%	10.7%	
	Q3	103	20.0%	17	17.5%	86	20.6%	3.1%	
	Q4	103	20.0%	29	29.9%	74	17.7%	−12.2%	
	Q5 most	102	19.8%	40	41.2%	62	14.9%	−26.4%	
		514		97		417			
(b) Obesity prevalence									
	All adults	n/a	19.0%	n/a	24.2%	n/a	17.9%	**6.3%**	*
	Male adults	n/a	12.8%	n/a	17.9%	n/a	11.6%	**6.3%**	*
	Female adults	n/a	25.6%	n/a	30.6%	n/a	24.4%	**6.2%**	*
	Young adults	n/a	7.6%	n/a	9.4%	n/a	7.1%	**2.3%**	*
	Adults	n/a	23.8%	n/a	29.9%	n/a	22.4%	**7.5%**	*
	Older adults	n/a	14.8%	n/a	23.3%	n/a	12.8%	**10.5%**	*

Note: Q = Quintile, n = number, % = proportion of column total, Urban = City, Rural = Regency. Data on district characteristics are from the World Bank and obesity data are from Basic Health Survey 2018. Bold numbers with asterisk (*) show statistically significance at 5% level (see Table A1 for the regression outputs).

**Table 3:** Ten districts with LOWEST prevalence of obesity among adults in Indonesia.

		Prevalence	Province	Region	Urban	Poverty	Education	Pop (000)
(a) All adults								
	Kab. Sumba Tengah	3.3%	East Nusa Tenggara	Papua	Rural	35%	44%	68
	Kab. Sumba Barat Daya	3.4%	East Nusa Tenggara	Papua	Rural	29%	42%	319
	Kab. Sabu Raijua	4.0%	East Nusa Tenggara	Papua	Rural	31%	69%	86
	Kab. Timor Tengah Selatan	4.3%	East Nusa Tenggara	Papua	Rural	28%	52%	459
	Kab. Manggarai Timur	5.0%	East Nusa Tenggara	Papua	Rural	27%	43%	272
	Kab. Nias	5.1%	North Sumatra	Sumatera	Rural	16%	62%	136
	Kab. Belu	5.3%	East Nusa Tenggara	Papua	Rural	16%	54%	206
	Kab. Sumba Barat	5.5%	East Nusa Tenggara	Papua	Rural	29%	55%	122
	Kab. Jayawijaya	5.7%	Papua	Papua	Rural	39%	67%	206
	Kab. Yahukimo	6.4%	Papua	Papua	Rural	39%	12%	181
	AVERAGE					**29%**	**50%**	**206**
(b) Male adults								
	Kab. Sumba Tengah	2%	East Nusa Tenggara	Papua	Rural	35%	44%	68
	Kab. Sabu Raijua	2%	East Nusa Tenggara	Papua	Rural	31%	69%	86
	Kab. Manggarai Timur	3%	East Nusa Tenggara	Papua	Rural	27%	43%	272
	Kab. Yahukimo	3%	Papua	Papua	Rural	39%	12%	181
	Kab. Sumba Barat Daya	3%	East Nusa Tenggara	Papua	Rural	29%	42%	319
	Kab. Jayawijaya	4%	Papua	Papua	Rural	39%	67%	206
	Kab Pesisir Barat	4%	Lampung	Sumatera	Rural	15%	72%	150
	Kab. Belu	3.9%	East Nusa Tenggara	Papua	Rural	16%	54%	206
	Kab. Nias	4.0%	North Sumatra	Sumatera	Rural	16%	62%	136
	Kab. Timor Tengah Selatan	4.0%	East Nusa Tenggara	Papua	Rural	28%	52%	459
	AVERAGE					**27%**	**52%**	**208**
(c) Female adults								
	Kab. Sumba Barat Daya	4%	East Nusa Tenggara	Papua	Rural	29%	42%	319
	Kab. Timor Tengah Selatan	5%	East Nusa Tenggara	Papua	Rural	28%	52%	459
	Kab. Sumba Tengah	5%	East Nusa Tenggara	Papua	Rural	35%	44%	68
	Kab. Sabu Raijua	6%	East Nusa Tenggara	Papua	Rural	31%	69%	86
	Kab. Nias	6.1%	North Sumatra	Sumatera	Rural	16%	62%	136
	Kab. Sintang	6.4%	West Kalimantan	Kalimantan	Rural	10%	45%	396
	Kab. Asmat	6.6%	Papua	Papua	Rural	27%	21%	88
	Kab. Sumba Barat	6.6%	East Nusa Tenggara	Papua	Rural	29%	55%	122
	Kab. Belu	6.7%	East Nusa Tenggara	Papua	Rural	16%	54%	206
	Kab. Manggarai Timur	7.1%	East Nusa Tenggara	Papua	Rural	27%	43%	272
	AVERAGE					**25%**	**49%**	**215**
(d) Young adults								
	Kab. Manggarai Timur	0%	East Nusa Tenggara	Papua	Rural	27%	43%	272
	Kab. Belu	0%	East Nusa Tenggara	Papua	Rural	16%	54%	206
	Kab. Sumba Tengah	1%	East Nusa Tenggara	Papua	Rural	35%	44%	68
	Kab. Jayawijaya	1%	Papua	Papua	Rural	39%	67%	206
	Kab. Timor Tengah Selatan	1%	East Nusa Tenggara	Papua	Rural	28%	52%	459
	Kab. Sumba Barat Daya	1%	East Nusa Tenggara	Papua	Rural	29%	42%	319
	Kab. Lanny Jaya	1%	Papua	Papua	Rural	40%	46%	172
	Kb. Manggarai	1%	East Nusa Tenggara	Papua	Rural	21%	51%	319
	Kab. Kupang	1%	East Nusa Tenggara	Papua	Rural	23%	58%	347
	Kab. Sabu Raijua	1%	East Nusa Tenggara	Papua	Rural	31%	69%	86
	AVERAGE					**29%**	**53%**	**246**
(e) Adults								
	Kab. Sumba Tengah	4.8%	East Nusa Tenggara	Papua	Rural	35%	44%	68
	Kab. Sumba Barat Daya	4.9%	East Nusa Tenggara	Papua	Rural	29%	42%	319
	Kab. Sabu Raijua	5.1%	East Nusa Tenggara	Papua	Rural	31%	69%	86
	Kab. Timor Tengah Selatan	5.4%	East Nusa Tenggara	Papua	Rural	28%	52%	459
	Kab. Jayawijaya	6.4%	Papua	Papua	Rural	39%	67%	206
	Kab. Nias	6.7%	North Sumatra	Sumatera	Rural	16%	62%	136
	Kab. Manggarai Timur	6.7%	East Nusa Tenggara	Papua	Rural	27%	43%	272
	Kab. Yahukimo	6.9%	Papua	Papua	Rural	39%	12%	181
	Kab. Asmat	7.0%	Papua	Papua	Rural	27%	21%	88
	Kab. Sumba Barat	7.0%	East Nusa Tenggara	Papua	Rural	29%	55%	122
	AVERAGE					**30%**	**47%**	**194**
(f) Older adults								
	Kab. Diyai	0.0%	Papua	Papua	Rural	43%	51%	69
	Kab. Mambramo Tengah	0.0%	Papua	Papua	Rural	37%	54%	46
	Kab. Nduga	0.0%	Papua	Papua	Rural	38%	9%	94
	Kab. Puncak Jaya	0.0%	Papua	Papua	Rural	36%	21%	115
	Kab. Intan Jaya	0.0%	Papua	Papua	Rural	43%	9%	46
	Kab. Lanny Jaya	0.0%	Papua	Papua	Rural	40%	46%	172
	Kab. Dogiyai	0.0%	Papua	Papua	Rural	30%	39%	92
	Kab. Paniayi	0.0%	Papua	Papua	Rural	37%	25%	164
	Kab. Yalimo	0.0%	Papua	Papua	Rural	35%	28%	59
	Kab. Waropen	0.3%	Papua	Papua	Rural	31%	61%	28
	AVERAGE					**37%**	**34%**	**89**

Note: Urban = City, Rural = Regency; Pop = Population. The districts are ordered by prevalence (column 1). Boldface values show the average.

**Table 4:** Ten districts with HIGHEST prevalence of obesity among adults in Indonesia, 2018.

		Prevalence	Province	Region	Urban	Poverty	Education	Pop (000)
(a) All adults								
	Kab. Yalimo	40.3%	Papua	Papua	Rural	35%	28%	59
	Kab. Karo	34.1%	North Sumatera	Sumatera	Rural	9%	74%	389
	Kota Tomohon	33.8%	North Sulawesi	Sulawesi	Urban	6%	71%	100
	Kota Jakarta Pusat	32.1%	Jakarta	Jawa	Urban	4%	55%	914
	Kab. Minahasa	31.7%	North Sulawesi	Sulawesi	Rural	7%	65%	329
	Kota Padang Sidempuan	31.6%	North Sumatera	Sumatera	Urban	8%	77%	210
	Kota Jakarta Timur	30.7%	Jakarta	Jawa	Urban	3%	67%	2827
	Kota Pematang Siantar	30.1%	North Sumatera	Sumatera	Urban	9%	77%	247
	Kab. Minahasa Selatan	30.0%	North Sulawesi	Sulawesi	Rural	9%	62%	205
	Kota Bitung	29.9%	North Sulawesi	Sulawesi	Urban	7%	57%	205
	AVERAGE					**10%**	**63%**	**548**
(b) Male adults								
	Kab. Yalimo	41.6%	Papua	Papua	Rural	35%	28%	59
	Kab. Puncak	30.9%	Papua	Papua	Rural	38%	9%	103
	Kota Tomohon	27.9%	North Sulawesi	Sulawesi	Urban	6%	71%	100
	Kab. Minahasa	26.8%	North Sulawesi	Sulawesi	Rural	7%	65%	329
	Kota Jakarta Pusat	25.8%	Jakarta	Jawa	Urban	4%	55%	914
	Kota Padang Sidempuan	25.6%	North Sumatera	Sumatera	Urban	8%	77%	210
	Kota Manado	25.5%	North Sulawesi	Sulawesi	Urban	5%	66%	425
	Kota Denpasar	25.5%	Bali	Jawa	Urban	2%	63%	879
	Kota Banda Aceh	25.2%	Aceh	Sumatera	Urban	7%	82%	250
	Kab. Karo	24.9%	North Sumatera	Sumatera	Rural	9%	74%	389
	AVERAGE					**12%**	**59%**	**366**
(c) Female adults								
	Kep Seribu	44.1%	Jakarta	Jawa	Rural	12%	71%	23
	Kab. Karo	43.2%	North Sumatera	Sumatera	Rural	9%	74%	389
	Kab Bener Meriah	42.7%	Aceh	Sumatera	Rural	20%	67%	137
	Kab Aceh Tengah	41.2%	Aceh	Sumatera	Rural	16%	73%	196
	Kota Tomohon	39.7%	North Sulawesi	Sulawesi	Urban	6%	71%	100
	Kab. Kep Talaud	39.7%	North Sulawesi	Sulawesi	Rural	10%	71%	89
	Kab. Minahasa Selatan	39.5%	North Sulawesi	Sulawesi	Rural	9%	62%	205
	Kota. Tidore Kepulauan	39.5%	North Maluku	Papua	Urban	6%	74%	97
	Kab. Yalimo	38.8%	Papua	Papua	Rural	35%	28%	59
	Kab. Manowari Selatan	38.6%	West Papua	Papua	Rural	31%	47%	22
	AVERAGE					**15%**	**64%**	**132**
(d) Young adults								
	Kab. Yalimo	38.0%	Papua	Papua	Rural	35%	28%	59
	Kab. Pegunungan Bintang	25.3%	Papua	Papua	Rural	31%	21%	72
	Kab. Waropen	22.9%	Papua	Papua	Rural	31%	61%	28
	Kab. Paniayi	18.7%	Papua	Papua	Rural	37%	25%	164
	Kota Samarinda	18.0%	East Kalimantan	Kalimantan	Urban	5%	66%	811
	Kab Tabanan	17.3%	Bali	Jawa	Rural	4%	81%	436
	Kab. Boven Digul	16.6%	Papua	Papua	Rural	20%	35%	63
	Kota Tomohon	16.6%	North Sulawesi	Sulawesi	Urban	6%	71%	100
	Kota Balikpapan	16.3%	East Kalimantan	Kalimantan	Urban	3%	69%	615
	Kota Madiun	16.3%	East Java	Jawa	Urban	4%	80%	175
	AVERAGE					**18%**	**54%**	**252**
(e) Adults								
	Kota Padang Sidempuan	42.1%	North Sumatera	Sumatera	Urban	8%	77%	210
	Kab. Yalimo	41.1%	Papua	Papua	Rural	35%	28%	59
	Kab. Karo	40.8%	North Sumatera	Sumatera	Rural	9%	74%	389
	Kota Tomohon	40.0%	North Sulawesi	Sulawesi	Urban	6%	71%	100
	Kota Lhokseumawe	38.2%	Aceh	Sumatera	Urban	12%	76%	191
	Kota Pematang Siantar	38.1%	North Sumatera	Sumatera	Urban	9%	77%	247
	Kota Blitar	37.9%	East Java	Jawa	Urban	7%	84%	138
	Kota Manado	37.8%	North Sulawesi	Sulawesi	Urban	5%	66%	425
	Kab. Mahakam Ulu	37.7%	East Kalimantan	Kalimantan	Rural	12%	52%	26
	Kab. Minahasa	37.1%	North Sulawesi	Sulawesi	Rural	7%	65%	329
	AVERAGE					**11%**	**67%**	**211**
(f) Older adults								
	Kota Banda Aceh	38.1%	Aceh	Sumatera	Urban	7%	82%	250
	Kep Seribu	37.9%	Jakarta	Jawa	Rural	12%	71%	23
	Kota Ternate	36.3%	North Maluku	Papua	Urban	3%	63%	213
	Kota Padang Sidempuan	35.5%	North Sumatera	Sumatera	Urban	8%	77%	210
	Kota Bekasi	35.3%	West Java	Jawa	Urban	4%	71%	2709
	Kota Tomohon	34.9%	North Sulawesi	Sulawesi	Urban	6%	71%	100
	Kota Medan	34.4%	North Sumatera	Sumatera	Urban	8%	62%	2209
	Kab. Karo	33.3%	North Sumatera	Sumatera	Rural	9%	74%	389
	Kab. Minahasa Utara	33.1%	North Sulawesi	Sulawesi	Rural	7%	61%	198
	Kota Jayapura	32.6%	Papua	Papua	Urban	11%	62%	283
	AVERAGE					**8%**	**69%**	**658**

Note: Urban = City, Rural = Regency; Pop = Population. The districts are ordered by prevalence (column 1). Boldface values show the average.

**Table 5:** Geographic and socioeconomic disparity in obesity among adults.

		All Districts (*n* = 514)						Urban (*n* = 97)						Rural (*n* = 417)					
		All	Male	Female	Young		Older	All	Male	Female	Young		Older	All	Male	Female	Young		Older
		Adults	Adults	Adults	Adults	Adults	Adults	Adults	Adults	Adults	Adults	Adults	Adults	Adults	Adults	Adults	Adults	Adults	Adults
		(1)	(2)	(3)	(4)	(5)	(6)	(7)	(8)	(9)	(10)	(11)	(12)	(13)	(14)	(15)	(16)	(17)	(18)
Region																			
	Papua	16.0%	11.6%	20.7%	5.8%	20.1%	11.5%	22.2%	15.7%	29.1%	7.6%	28.8%	22.6%	15.4%	11.2%	19.9%	5.6%	19.2%	10.3%
	Sulawesi	19.4%	13.0%	25.8%	7.6%	24.3%	15.8%	23.9%	18.6%	29.2%	9.6%	30.1%	24.1%	18.6%	12.1%	25.3%	7.2%	23.3%	14.5%
	Kalimantan	18.6%	12.6%	25.3%	8.0%	22.9%	12.5%	23.5%	17.6%	29.9%	10.7%	28.7%	18.0%	17.6%	11.6%	24.4%	7.5%	21.8%	11.5%
	Sumatera	19.5%	12.5%	27.0%	7.1%	24.5%	16.5%	23.6%	17.1%	30.3%	8.4%	29.4%	24.1%	18.4%	11.2%	26.1%	6.7%	23.2%	14.4%
	Java	20.7%	14.0%	27.4%	9.3%	25.7%	15.5%	25.4%	19.1%	31.8%	10.5%	30.8%	23.8%	18.9%	12.1%	25.8%	8.9%	23.8%	12.3%
	Absolute	**4.7%**	2.4%	**6.7%**	**3.5%**	**5.6%**	4.0%	3.2%	3.4%	2.7%	2.9%	2.0%	1.2%	**3.5%**	0.9%	**5.9%**	**3.3%**	**4.6%**	2.0%
	Relative	**1.29**	1.21	**1.32**	**1.60**	**1.28**	1.35	1.14	1.22	1.09	1.38	1.07	1.05	**1.23**	1.08	**1.30**	**1.59**	**1.24**	1.19
Income																			
	Q1 poor	16.3%	11.3%	21.5%	6.5%	20.3%	10.8%	18.3%	13.3%	23.3%	6.0%	23.3%	23.0%	16.2%	11.2%	21.5%	6.5%	20.3%	10.4%
	Q2	17.1%	10.7%	23.6%	6.4%	21.7%	12.7%	23.9%	16.5%	31.8%	8.4%	29.8%	21.4%	16.7%	10.4%	23.2%	6.3%	21.2%	12.3%
	Q3	19.6%	12.7%	26.8%	8.1%	24.5%	15.0%	22.8%	17.0%	28.9%	8.0%	28.9%	21.8%	19.2%	12.1%	26.5%	8.1%	23.9%	14.0%
	Q4	20.1%	13.5%	27.0%	7.7%	25.2%	16.5%	24.1%	18.2%	30.0%	8.8%	30.2%	24.5%	19.1%	12.3%	26.2%	7.5%	23.9%	14.4%
	Q5 rich	22.1%	15.7%	28.8%	9.2%	27.1%	18.7%	24.9%	18.4%	31.5%	10.3%	30.3%	23.3%	19.0%	12.7%	25.9%	7.9%	23.6%	13.7%
	Absolute	**5.8%**	**4.4%**	**7.3%**	**2.7%**	**6.8%**	**7.9%**	**6.6%**	5.1%	**8.2%**	4.3%	**7.0%**	0.3%	**2.8%**	1.5%	**4.4%**	1.4%	**3.3%**	**3.3%**
	Relative	**1.36**	**1.39**	**1.34**	**1.42**	**1.33**	**1.73**	**1.36**	1.38	**1.35**	1.72	**1.30**	1.01	**1.17**	1.13	**1.20**	1.22	**1.16**	**1.32**
Education																			
	Q1 least	16.0%	10.6%	21.7%	6.7%	19.8%	10.1%	n/a	n/a	n/a	n/a	n/a	n/a	16.0%	10.6%	21.7%	6.7%	19.8%	10.1%
	Q2	18.0%	11.7%	24.6%	7.5%	22.5%	13.2%	24.1%	18.5%	29.8%	10.2%	29.5%	21.3%	17.2%	10.8%	23.9%	7.1%	21.7%	12.3%
	Q3	19.4%	13.0%	26.2%	7.5%	24.4%	15.2%	23.1%	16.6%	29.8%	8.9%	28.5%	22.6%	18.7%	12.2%	25.5%	7.2%	23.6%	13.8%
	Q4	20.3%	13.6%	27.3%	7.9%	25.2%	17.1%	23.9%	17.4%	30.6%	9.8%	29.2%	23.1%	18.9%	12.2%	26.0%	7.2%	23.7%	14.7%
	Q5 most	21.5%	15.1%	28.1%	8.2%	27.0%	18.2%	24.8%	18.7%	31.1%	9.2%	31.0%	24.2%	19.4%	12.7%	26.2%	7.6%	24.4%	14.3%
	Absolute	**5.5%**	**4.5%**	**6.4%**	1.5%	**7.2%**	**8.1%**	0.7%	0.2%	1.3%	−1.0%	1.5%	2.9%	**3.4%**	**2.1%**	**4.5%**	0.9%	**4.6%**	**4.2%**
	Relative	**1.34**	**1.42**	**1.29**	1.22	**1.36**	**1.80**	1.03	1.01	1.04	0.90	1.05	1.14	**1.21**	**1.20**	**1.21**	1.13	**1.23**	**1.42**

Note: Q = Quintile; Java region includes Bali; Papua region includes Maluku and Nusa Tenggara. Income quintile used district-level poverty rate (e.g., Q1 = 20% of districts with highest poverty rate). Absolute (Relative) = Difference (Ratio) between Papua and Java as well as Q1 and Q5. For education, absolute (relative) was between Q1 and Q5 except among urban (Q2 and Q5). Boldface values show statistically significance at 5% level (see Table A2 for the regression outputs).

## Data Availability

The data presented in this study are available on request from the corresponding author. The data are not publicly available due to local policy.

## Appendix A

**Table A1:** Regression outputs for urban/rural differences.

	All	Males	Females	Young Adults	Adults	Older Adults
	Coef	Coef	Coef	Coef	Coef	Coef
Rural	Reference					
Urban	6.31 **	6.32 **	6.16 **	2.29 **	7.51 **	10.49 **
Constant	17.85 **	11.59 **	24.41 **	7.15 **	22.36 **	12.79 **
Observations	514	514	514	514	514	514
R-squared	0.20	0.23	0.12	0.06	0.20	0.32

Note: Coef = OLS Coefficient; Significance level ** *p* < 0.01

**Table A2:** Regression outputs for geographic and socioeconomic disparity in obesity.

	All	Males	Females	Young Adults	Adults	Older Adults
	Coef	Coef	Coef	Coef	Coef	Coef
(a) All districts (N = 514)						
Papua	Reference					
Java	1.94 *	0.12	3.53 **	2.85 **	2.14 *	−0.72
Sumatera	0.92	−1.34	3.27 **	0.76	0.98	0.59
Kalimantan	−0.73	−1.90 *	0.81	1.24	−1.12	−4.72 **
Sulawesi	2.01 *	0.51	3.33 **	1.65 **	2.32 *	1.61
Income						
Quintile 1 poor	Reference					
Quintile 2	−0.54	−1.12	0.06	−0.93	−0.35	0.82
Quintile 3	2.09 **	1.17	3.03 **	0.36	2.68 **	4.03 **
Quintile 4	2.66 **	2.07 **	3.30 **	0.16	3.44 **	5.48 **
Quintile 5 rich	4.62 **	4.10 **	5.23 **	1.39 *	5.31 **	8.34 **
Education						
Quintile 1 least	Reference					
Quintile 2	0.82	0.49	1.11	0.31	1.35	1.09
Quintile 3	2.51 **	2.03 **	2.94 **	0.52	3.50 **	3.29 **
Quintile 4	3.16 **	2.55 **	3.70 **	0.83	4.09 **	4.89 **
Quintile 5 most	3.96 **	3.88 **	3.81 **	0.97	5.33 **	5.23 **
(b) Urban (N = 97)						
Papua	Reference					
Java	2.39	3.12	1.42	2.17	1.23	0.05
Sumatera	0.94	1.10	0.63	0.53	0.10	1.21
Kalimantan	0.64	2.04	−0.54	2.14	−0.36	−5.47
Sulawesi	1.02	2.75	−0.93	1.22	0.80	0.66
Income						
Quintile 1 poor	Reference					
Quintile 2	5.09	2.13	8.56 *	2.26	5.98	−1.39
Quintile 3	4.51	3.45	5.73	2.20	5.57	−1.28
Quintile 4	4.97	3.25	6.78 *	2.05	6.32 *	2.15
Quintile 5 rich	6.03 *	3.66	8.54 **	3.55	6.77 *	1.73
Education						
Quintile 1 least	n/a	n/a	n/a	n/a	n/a	n/a
Quintile 2	Reference					
Quintile 3	−0.70	−1.39	0.07	−0.97	−0.72	1.47
Quintile 4	0.50	−0.34	1.45	0.36	0.42	1.55
Quintile 5 most	1.38	1.23	1.51	−0.09	2.06	2.18
(c) Rural (N = 417)						
Papua	Reference					
Java	2.00 *	−0.14	3.91 **	3.05 **	2.64 *	−0.73
Sumatera	1.35	−1.28	4.12 **	0.91	1.69	1.21
Kalimantan	0.29	−1.14	2.08	1.55	0.24	−2.28
Sulawesi	2.44 **	0.51	4.22 **	1.74 **	2.91 **	2.22 *
Income						
Quintile 1 poor	Reference					
Quintile 2	−0.85	−1.18	−0.55	−1.09	−0.76	0.77
Quintile 3	1.66 *	0.81	2.53 *	0.16	1.96 *	3.53 **
Quintile 4	1.81 *	1.23	2.43 *	−0.09	2.27 *	3.72 **
Quintile 5 rich	2.21 *	1.85	2.75 *	0.25	2.53 *	4.29 **
Education						
Quintile 1 least	Reference					
Quintile 2	0.60	0.19	0.96	0.23	1.06	0.90
Quintile 3	2.26 **	1.80 **	2.69 **	0.53	3.23 **	2.47 **
Quintile 4	2.44 **	1.75 *	3.08 **	0.46	3.27 **	3.50 **
Quintile 5 most	2.68 **	2.54 **	2.58 *	0.69	3.60 **	2.81 **

Note: Coef = OLS Coefficient. In each panel (a–c), each column provides each multivariate OLS regression output (command-regress-in STATA 15). Significance level ** *p* < 0.01, * *p* < 0.05.
